# Genetic relationships among Italian and Mexican maize-rhizosphere *Burkholderia cepacia *complex (BCC) populations belonging to *Burkholderia cenocepacia *IIIB and BCC6 group

**DOI:** 10.1186/1471-2180-11-228

**Published:** 2011-10-13

**Authors:** Annamaria Bevivino, Barbara Costa, Cristina Cantale, Silvia Cesarini, Luigi Chiarini, Silvia Tabacchioni, Jesus Caballero-Mellado, Claudia Dalmastri

**Affiliations:** 1 ENEA (Italian National Agency for New Technologies, Energy and Sustainable Development) Casaccia Research Center - Technical Unit for Sustainable Development and Innovation of Agro-Industrial System, Via Anguillarese 301, 00123 S. Maria di Galeria, Rome, Italy; 2 Centro de Ciencias Genómicas, Universidad Nacional Autónoma de México, Ap. Postal 565-A, Cuernavaca, Morelos, Mexico

## Abstract

**Background:**

A close association between maize roots and *Burkholderia cepacia *complex (BCC) bacteria has been observed in different locations globally. In this study we investigated by MultiLocus Restriction Typing (MLRT) the genetic diversity and relationships among *Burkholderia cenocepacia *IIIB and BCC6 populations associated with roots of maize plants cultivated in geographically distant countries (Italy and Mexico), in order to provide new insights into their population structure, evolution and ecology.

**Results:**

The 31 *B. cenocepacia *IIIB and 65 BCC6 isolates gave rise to 29 and 39 different restriction types (RTs), respectively. Two pairs of isolates of *B. cenocepacia *IIIB and BCC6, recovered from both Italian and Mexican maize rhizospheres, were found to share the same RT. The eBURST (Based Upon Related Sequence Types) analysis of MLRT data grouped all the *B. cenocepacia *IIIB isolates into four clonal complexes, with the RT-4-complex including the 42% of them, while the majority of the BCC6 isolates (94%) were grouped into the RT-104-complex. These two main clonal complexes included RTs shared by both Italian and Mexican maize rhizospheres and a clear relationship between grouping and maize variety was also found. Grouping established by eBURST correlated well with the assessment using unweighted-pair group method with arithmetic mean (UPGMA). The standardized index of association values obtained in both *B. cenocepacia *IIIB and BCC6 suggests an epidemic population structure in which occasional clones emerge and spread.

**Conclusions:**

Taken together our data demonstrate a wide dispersal of certain *B. cenocepacia *IIIB and BCC6 isolates in Mexican and Italian maize rhizospheres. Despite the clear relationship found between the geographic origin of isolates and grouping, identical RTs and closely related isolates were observed in geographically distant regions. Ecological factors and selective pressure may preferably promote some genotypes within each local microbial population, favouring the spread of a single clone above the rest of the recombinant population.

## Background

The *Burkholderia cepacia *complex (BCC) is an ubiquitous and extremely versatile group of closely related Gram-negative bacteria, currently divided into 17 species [1,2]. BCC bacteria emerged in the 1980s as opportunistic human pathogens responsible for devastating lung infections in people with cystic fibrosis and chronic granulomatous disease [3]. BCC has also been shown to colonise natural habitats including agricultural soils, plant rhizospheres, and river waters [4-7]. The maize rhizosphere is a favourable niche for BCC bacteria, probably due to their ability to metabolise at high rates maize root exudates [8] and has also been suggested to represent a natural reservoir of bacterial strains that may exhibit pathogenic traits [9-13]. A close association between maize roots and BCC has been observed in a number of different locations worldwide [6,14-17]. Studies on BCC populations recovered from Italian maize rhizosphere have shown the presence of several BCC species such as *B. cepacia, B. cenocepacia *(*recA *lineage IIIB), *B. ambifaria, B. pyrrocinia*, and BCC groups such as BCC5 and BCC6 suggesting possible novel plant associated species within the complex [14,18-20]. In Mexico, where maize has traditionally been cultivated for thousands of years, *B. cenocepacia *(*recA *lineage IIIB) and *B. vietnamiensis *were isolated with other *Burkholderia *species from the rhizosphere of local and commercial varieties of maize plants cultivated in distant geographical regions [[21,22], our unpublished data]. 

The maize rhizosphere is a dynamic and active environment in which many factors may affect the diversity and activity of microbial communities [23,24]. The distribution of identical clones among BCC populations recovered from geographically disparate Italian maize rhizospheres suggested that bacterial flow may occur among BCC populations of different geographic areas [20]. Therefore, assessing the diversity of maize-rhizosphere associated BCC species in different and distant countries may provide critical insight into the population structure, evolution and ecology of such BCC populations. Indexing allelic variation in sets of housekeeping genes provides a good basis for estimating overall levels of genotypic variation in microbial populations [25,26]. Methods based on this principle, such as multilocus restriction typing (MLRT), multilocus enzyme electrophoresis (MLEE), and multilocus sequence typing (MLST), provide good insights into the genetic relationships among strains [27-30]. During the last decade, MLST has emerged as a powerful tool in studies of BCC epidemiology and population structure [31]. MLRT has a lower discrimination power than MLST, but acceptable turnaround time and lower cost make it really advantageous, especially for an 'in-house' initial genotype screening of isolates collected in large-scale [32-34]. Furthermore, MLRT has been used to study the global epidemiology and the population structure of *B. cenocepacia *[26,32], *Streptococcus pneumoniae *[28] and *Helicobacter pylori *[35], as well as to determine the genetic relationships among strains of *Neisseria meningitidis *[25,36], *Staphylococcus aureus *[37], *Escherichia coli *[38] and *Yersinia enterocolitica *biovar 1A [30]. The successful application of MLRT in these studies and the excellent correlation with MLST results [36,37] demonstrate its wide range of potential applications.

In the current study, we investigated the genetic relationships in *B. cenocepacia *IIIB and BCC6 populations associated with roots of maize plants cultivated in two distant countries (Italy and Mexico). Assessment was carried out by applying the MLRT scheme specifically developed for *B. cenocepacia *[26] also to BCC6 group, since it includes bacteria previously assigned to *B. cenocepacia *by means of *recA *polymorphism based tests [19,20]. We focused on *B. cenocepacia *IIIB as it is widely spread in both Italian and Mexican rhizospheres [[20,22], our unpublished data], besides its importance as an opportunistic pathogen in patients with cystic fibrosis [39], and on the underappreciated BCC6 group as it has only been isolated from Italian maize rhizosphere [20], although its real distribution has most likely been masked by *B. cenocepacia *IIIB. As the maize historically originates from Mexico, we have chosen to compare representatives isolates of our Italian *B. cenocepacia *IIIB and BCC6 collections with Mexican ones in order to provide new insights into maize-rhizosphere bacterial populations. In particular, we aimed to (i) describe the genetic structure of bacterial populations by evaluating the extent of linkage equilibrium between the different loci, (ii) assess whether the geographic origin of isolated bacteria influences the extent of their genetic diversity, and (iii) individuate the genetic similarities among the restriction types of *B. cenocepacia *IIIB and BCC6 group.

## Results

### RTs distribution among maize-rhizosphere BCC populations

**Figure 1:**
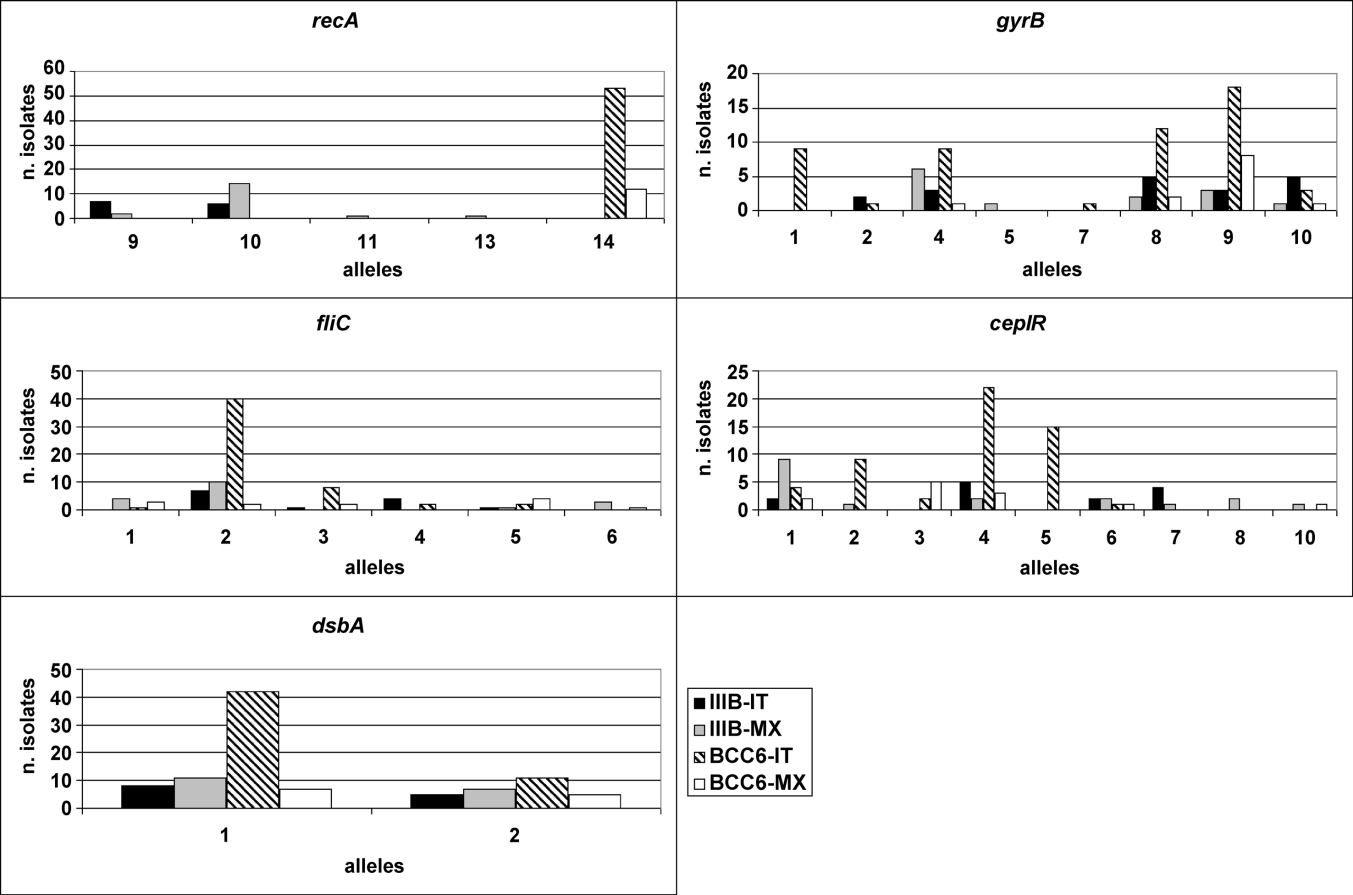
**Frequency of alleles among the 5 loci examined**. For each locus, the no. of times each allele occurs in both Italian and Mexican *B. cenocepacia *and BCC6 populations is shown.

For each of the five loci (*recA*, *gyrB*, *fliC*, *cepIR *and *dsbA*), amplified products of the expected size were obtained in each of the 96 BCC isolates (Tables 1 and 2). The number of different alleles present per locus in the *B. cenocepacia *IIIB population included: 4 (*recA*), 6 (*gyrB*), 6 (*fliC*), 7 (*cepIR*), and 2 (*dsbA*). While in the BCC6 population this differed slightly: 1 (*recA*), 7 (*gyrB*), 6 (*fliC*), 7 (*cepIR*), and 2 (*dsbA*). The frequency of each allele within each bacterial population is shown in Figure 1. In the *B. cenocepacia *IIIB population, *gyrB *and *cepIR *loci showed the highest diversity (*h *= 0.8108 and *h *= 0.8000, respectively), while *dsbA *and *recA *loci showed the lowest diversity (*h *= 0.4903 and *h *= 0.5140, respectively); in the BCC6 population, *cepIR *and *gyrB *loci showed a high diversity (*h *= 0.7702 and *h *= 0.7582, respectively), while no polymorphism was observed within *recA *locus (*h *= 0.0000). The mean genetic diversity (*H*_*mean*_) was 0.6576 ± 0.0680 for all *B. cenocepacia *IIIB isolates and 0.4918 * ± *0.1427 for all BCC6 isolates (Table 3).

**Table 1:** Restriction types (RTs) and eBURST grouping of Italian and Mexican maize-rhizosphere *B. cenocepacia *IIIB isolates.

RT	**Isolate name**^***a***^	**RFLP type**^***b***^	**IIIB-specific PCR**^***c***^	Alleles at the following loci				
				*recA*	*gyrB*	*fliC*	*cepIR*	*dsbA*
Group 1								
74	MDII-143p	I	+	9	10	2	4	1
112	MDII-151p	I	+	9	8	2	4	1
								
Group 2								
100	MDIII-B604	I	+	9	9	4	4	1
17	MexII-968	I	+	9	9	1	4	1
							
Group 3 (RT-4-complex)								
115	MDIII-B752	J'	+	10	9	2	7	1
21	MDIII-P378	J'	+	10	4	2	1	1
21	MexII-864	J'	+	10	4	2	1	1
3	MexII-57	J'	+	10	10	2	1	2
7	MexII-60	J'	+	10	10	2	8	2
7	MexII-857	J'	+	10	10	2	8	2
6	MexII-815	J'	+	10	10	2	1	1
53	MexII-845	J'	+	10	8	1	1	1
31	MexII-940	J'	+	10	9	1	1	1
36	MexII-945	J'	+	10	4	2	6	1
4*	MexII-967	J'	+	10	9	2	1	1
67	MexII-974	J'	+	10	8	2	6	1
57	MexII-994	J'	+	10	8	2	1	2
								
Group 4								
50	MexII-867	uk^*d*^	+	11	8	6	1	1
46	MexII-976	I	+	9	8	6	1	1
								
Singletons								
80	MCII-168	J'	+	10	5	3	6	2
27	MDII-129r	I	+	9	4	2	6	2
75	MDIII-B250	J'	+	10	8	4	4	1
114	MDIII-B716	I	+	9	4	2	7	1
28	MDIII-P410	I	+	9	4	5	7	2
22	MDIII-P461	J'	+	10	4	2	7	2
29	MDIII-T228	I	+	9	4	4	1	1
103	MDIII-T521	J'	+	10	9	4	4	2
70	MexII-206	uk^*e*^	+	13	10	5	7	2
47	MexII-264	J'	+	10	2	1	2	2
48	MexII-828	J'	+	10	2	2	4	1
33	MexII-863	J'	+	10	4	6	10	2

* The asterisk denotes the ancestral clone of the clonal complex.

^*a *^Italian isolates: MCII, MCIII, MVP-C1, MVP-C2, MDII, MDIII-B, MDIII-P, MDIII-T, followed by the number of isolate. Mexican isolates: MexII, followed by the number of isolate.

^*b *^*recA *RFLP with *Hae*III.

^*c *^The presence and absence of specific PCR amplification for *B. cenocepacia *IIIB is indicated by a signus "+" and "-", respectively.

^*d, e *^uk, unknown RFLP profiles: restriction profiles never recovered among BCC reference strains examined [56].

**Table 2:** Restriction types (RTs) and eBURST grouping of Italian and Mexican maize-rhizosphere BCC6 isolates.

RT	**Isolate name**^***a***^	**RFLP type**^***b***^	**IIIB-specific PCR**^***c***^	Alleles at the following loci				
				*recA*	*gyrB*	*fliC*	*cepIR*	*dsbA*
Group 1 (RT-104-complex)								
20	MexII-195	AD	+	14	9	5	4	2
26 (3)^*d*^	MCII-88^*e*^	AD	+	14	1	3	2	1
34 (8)^*d*^	MVP-C2-23^*e*^	AD	-	14	9	2	5	1
35 (2)^*d*^	MVP-C2-60^*e*^	AD	+	14	8	2	5	1
37 (2)^*d*^	MDII-107r^*e*^	AD	-	14	4	2	5	1
40	MVP-C2-21	AD	-	14	9	2	5	2
54	MexII-989	AD	+	14	4	5	4	1
55 (2)^*d*^	MDIII-T18^*e*^	AD	+	14	8	3	4	1
56	MexII-992	AD	-	14	8	2	1	2
58	MexII-1011	AD	-	14	9	1	6	1
59 (2)^*d*^	MexII-1005^*e*^	AD	+	14	9	1	3	2
60 (2)^*d*^	MexII-983^*e*^	AD	-	14	9	5	3	1
72	MCII-179	AD	+	14	1	4	2	1
77	MCII-13	AD	+	14	1	4	2	1
79 (2)^*d*^	MVP-C2-81^*e*^	AD	-	14	9	2	3	1
81 (6)^*d*^	MVP-C1-16^*e*^	AD	-	14	8	2	4	1
82 (3)^*d*^	MVP-C2-2^*e*^	AD	-	14	9	2	4	1
84	MDIII-P152	AD	-	14	9	3	4	2
85	MDIII-P253	AD	+	14	4	3	4	1
86	MDIII-P292	AD	-	14	8	5	1	1
87	MVP-C1-15	AD	-	14	8	2	4	2
88	MDIII-P3	AD	+	14	9	1	4	1
89	MCII-23	AD	+	14	2	2	5	1
95 (2)^*d*^	MCII-35^*e*^	AD	+	14	1	2	2	2
96	MCII-134	AD	+	14	1	3	2	2
97	MVP-C2-1	AD	+	14	7	2	4	2
98 (2)^*d*^	MDII-125r^*e*^	AD	+	14	10	2	4	1
99	MDII-103r	AD	-	14	9	3	4	1
104*	MDII-105r	AD	+	14	4	2	4	1
104*	MVP-C1-79	AD	-	14	4	2	4	1
106 (2)^*d*^	MDII-144p^*e*^	AD	+	14	4	2	4	2
110	MVP-C2-84	AD	-	14	4	2	2	2
111	MVP-C2-67	AD	-	14	8	2	1	1
113	MDII-110r	AD	-	14	10	2	5	1
116	MDIII-T258	AD	+	14	4	2	1	1
122	MexII-858	AD	-	14	9	2	3	2
Singletons								
19	MexII-125	AD	-	14	9	6	1	1
38	MDIII-T109	AD	+	14	9	4	1	2
61	MexII-831	AD	-	14	10	3	10	1
76	MCII-182	AD	+	14	1	5	6	1

^a, b, c, * ^see legend Table 1.

^*d *^In parenthesis, no. of isolates with same RT. RT26 (MCII-88, MCIII-CA-1, MCIII-CC-35); RT34 (MVP-C2-23, MVP-C2-53, MVP-C2-57, MVP-C2-63, MVP-C2-64, MVP-C2-76, MVP-C2-82, MDII-116r); RT35 (MVP-C2-60, MVP-C2-62); RT 37 (MDII-107r, MVP-C2-58); RT55 (MDIII-T18, MexII-829); RT59 (MexII-1005, MexII-1006); RT60 (MexII-983, MexII-984); RT79 (MVP-C2-81, MVP-C2-90); RT81 (MVP-C1-16, MVP-C1-21, MVP-C1-22, MVP-C1-78, MVP-C2-18, MDIII-P41); RT82 (MVP-C2-2, MDIII-B659, MDIII-P115); RT95 (MCII-35, MCII-36); RT98 (MDII-125r, MVP-C2-121p); RT106 (MDII-144p, MDIII-T301).

^*e *^representative isolate of RT.

**Table 3:** Linkage disequilibrium analysis of *B. cenocepacia *IIIB and BCC6 populations according to their geographic origin.

Group selection	Mean genetic **diversity (*H*_*mean*_)**^***a***^	Observed variance (V_D_)	Expected variance (V_e_)	$I_{A}^{S}$	***P *value**^***b***^	Linkage disequilibrium
***B. cenocepacia *IIIB population**						
						
All isolates	0.6576 ± 0.0680	1.1538	1.0332	0.0292	0.0187	Yes
RTs only	0.6675 ± 0.0671	1.0982	1.0196	0.0193	0.127	No
						
Italian isolates	0.6462 ± 0.0533	1.0629	1.0865	-0.0054	1.000	No
RTs only	0.6462 ± 0.0533	1.0629	1.0865	-0.0054	1.000	No
						
Mexican isolates	0.6235 ± 0.0776	1.3282	1.0534	0.0652	0.0041	Yes
RTs only	0.6250 ± 0.0760	1.2806	1.0565	0.0530	0.0323	Yes

**BCC6 population**						
						
All isolates	0.4918 ± 0.1427	0.9421	0.8423	0.0296	0.0025	Yes
RTs only	0.5447 ± 0.1499	0.7382	0.7906	-0.0165	1.000	No
						
Italian isolates	0.4518 ± 0.1425	0.9750	0.8324	0.0428	0.0002	Yes
RTs only	0.5195 ± 0.1477	0.7664	0.8118	-0.0140	1.000	No
						
Mexican isolates	0.5424 ± 0.1483	0.9159	0.8014	0.0357	0.164	No
RTs only	0.5778 ± 0.1573	0.6465	0.7249	-0.0271	1.000	No

^*a *^Mean genetic diversity per locus ± standard deviation.

^*b *^The measure of linkage disequilibrium is performed by testing the null hypothesis (H_O_):V_*D *_= V_*e*_, where V_*D *_is the variance calculated from the distribution of mismatch values of variance and V_*e *_is the variance expected for linkage equilibrium. *P *values are derived from parametric method [57] and indicate the significance of linkage disequilibrium. If the $I_{A}^{S}$ (*P *< 0.05) value differs significantly from zero, the null hypothesis of linkage equilibrium is rejected.

A restriction type (RT) for each isolate was generated by combining information for each of the five loci. MLRT divided the 31 *B. cenocepacia *IIIB and the 65 BCC6 isolates into 29 and 39 different RTs, respectively (Tables 1 and 2). Some BCC isolates from Mexico and Italy were found to share identical RTs; i.e., RT-21 was shared by two *B. cenocepacia *IIIB isolates (MDIII-P378 and MexII-864) and RT-55 was shared by two BCC6 isolates (MDIII-T18 and MexII-829). Many RTs were found to type more than one isolate within the Italian BCC6 population (RT 26, RT 34, RT 35, RT 37, RT 79, RT 81, RT 82, RT 95, RT 98, RT 104, RT 106) and the Mexican BCC6 population (RT 59, RT 60) (Table 2). This was also seen in the case of one RT in the *B. cenocepacia *IIIB population (RT 7) (Table 1).

### Genetic relationships among isolates

Using the eBURST algorithm, clonal complexes or closely related RTs were defined as groups in which each isolate is identical to at least one other isolate at four of the five loci. In addition, within each major clonal complex, the putative ancestral genotype was defined as the RT that differs from the largest number of other RTs at only a single locus, and the single-locus variants (SLVs) as the RTs that differ from the ancestral genotype at only one locus. RTs which differ from all other RTs at more than two loci were designated as singleton RTs.

**Figure 2:**
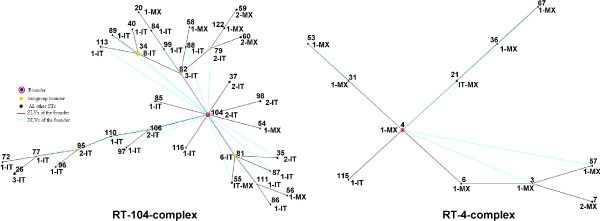
**Schematic representation of the two major clonal complexes: RT-104-complex (BCC6 population) and RT-4-complex (*B. cenocepacia *IIIB population)**. Each number represents a restriction type (RT). Data are presented as burst diagrams obtained using the eBURST algorithm v3: the primary founder or ancestral genotype (blue) is defined as the RT that differs from the largest number of other RTs within the complex at only one locus, i.e. the RT that has the greatest number of single-locus variants (SLVs); linked single-locus variants (SLVs) (purple) are the RTs differing from the ancestral genotype at one locus; linked double-locus variants (DLVs) (cyan) are the RTs differing from the ancestral genotype at two loci; the subgroup founder (yellow) is the RT that appears to have diversified to produce its own SLVs, i.e. a RT with at least two assigned descendent SLVs.

Within the *B. cenocepacia *IIIB population, 19 isolates (61%) were distinguished by 15 RTs and grouped into four clonal complexes, while the remaining 12 isolates (8 Italian and 4 Mexican) were characterized as singleton RTs. RT-4-complex, with RT4 (typing one Mexican isolate) as its putative ancestral genotype, represented the major clonal complex since it included 42% of isolates (11 Mexican and 2 Italian isolates), with RT 115 (one Italian isolate), RT 21 (one Mexican and one Italian isolates), RT 31 (one Mexican isolate), and RT 6 (one Mexican isolate) as SLVs of the predicted primary founder. The other three clonal complexes included few isolates and then may be considered as doublets of RTs (Table 1 and Figure 2). As far as the BCC6 group is concerned, the eBURST algorithm grouped most of the BCC6 isolates (94%) into one clonal complex, designated RT-104-complex, with RT104 (typing two Italian isolates) as putative ancestral genotype, while four isolates (two Italian and two Mexican) were branded as four singleton RTs. The RT-104-complex included 35 RTs (typing 51 Italian and 10 Mexican isolates), with RT54 (typing one Mexican isolate) and RT 37, RT 82, RT85, RT98, RT106 and RT116 (typing Italian isolates) as SLVs of the predicted primary founder (Table 2 and Figure 2).

**Figure 3:**
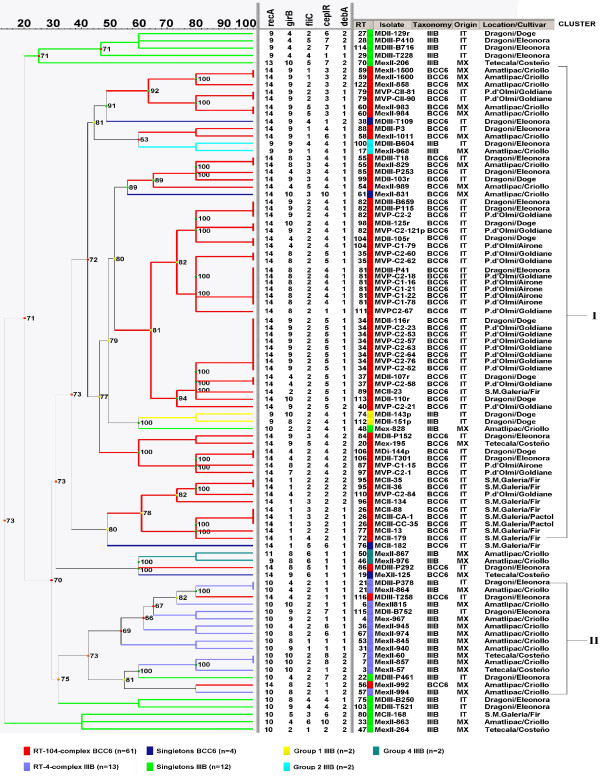
**UPGMA dendrogram generated by BioNumerics software showing the genetic relationships among all *B. cenocepacia *IIIB and BCC6 isolates**. The cophenetic correlation coefficient is shown at each branch, together with a coloured dot, of which the colour ranges between green-yellow-orange-red according to decreasing cophenetic correlation. The Cluster Cutoff method was applied to define the most reliable clusters. The branches found below the cutoff values are shown in dashed lines. Data concerning *B. cenocepacia *and BCC6 isolates are also included.

The genetic relationships among isolates belonging to the major complexes of *B. cenocepacia *IIIB and BCC6 populations (RT-4-complex and RT-104-complex, respectively) as well as to the other minor complexes and singletons are shown in Figure 3. The dendrogram constructed using the UPGMA algorithm in BioNumerics revealed that all isolates were grouped in two main clusters, corresponding to the major eBURST clonal complexes. The major cluster (I) included the BCC6 RT-104 clonal complex, while the cluster II comprised the *B. cenocepacia *IIIB RT-4 clonal complex. Interestingly, within the cluster I, which mostly comprised the BCC6 isolates, the *B. cenocepacia *IIIB eBURST groups 1 and 2 were also present, while two BCC6 isolates (MDIII-T258 and MexII-992) belonging to the RT-104 clonal complex fell within the cluster II which mostly included *B. cenocepacia *IIIB isolates.

### Standardized index of association ($I_{A}^{S}$) and population structure

Evidence for recombination and clonality in *B. cenocepacia *IIIB and BCC6 rhizosphere populations was assessed using standardized index of association ($I_{A}^{S}$). A $I_{A}^{S}$ value differing from zero characterizes clonal population (linkage disequilibrium), while a $I_{A}^{S}$ value close to zero characterizes freely recombining population (linkage equilibrium). $I_{A}^{S}$ values including all rhizosphere isolates or single representatives of each RT were calculated separately to put in evidence bias due to epidemic structure for (i) the entire *B. cenocepacia *IIIB population, (ii) the Italian *B. cenocepacia *IIIB population, (iii) the Mexican *B. cenocepacia *IIIB population, (iv) the entire BCC6 population, (v) the Italian BCC6 population, and (vi) the Mexican BCC6 population (Table 3). In the *B. cenocepacia *IIIB population, the value of $I_{A}^{S}$ calculated considering all 31 isolates differed significantly from zero ($I_{A}^{S}=0.0292$; *P *= 0.0187) indicating a high level of linkage disequilibrium and a non-random association among alleles at different loci. $I_{A}^{S}$ decreased when only single representatives of each RT were included ($I_{A}^{S}=0.0193$; *P *= 0.127), suggesting a random association between alleles in some subgroups (linkage equilibrium). In the BCC6 population, the $I_{A}^{S}$ value calculated including all 65 isolates was 0.0296 (*P *= 0.025), indicating a linkage disequilibrium which disappeared when the analysis was repeated with each RT treated as an individual (*P *> 0.05), suggesting a possible epidemic population structure in which occasional clones emerge and spread.

Considering each bacterial population according to its geographic origin, a random association among the alleles (linkage equilibrium) within the Italian *B. cenocepacia *IIIB population was found either when all isolates or each RT treated as an individual were considered (*P *> 0.05); conversely, the Mexican *B. cenocepacia *IIIB population showed linkage disequilibrium at both levels (Table 3). Linkage disequilibrium was also observed within the Italian BCC6 population when all 53 isolates were considered ($I_{A}^{S}=0.0428$; *P *= 0.0002); conversely, when the analysis was restricted to RTs taken as units, linkage equilibrium was found ($I_{A}^{S}=-0.0140$; *P *> 0.05). Within the Mexican BCC6 maize rhizosphere population, linkage equilibrium was found either when all isolates or RTs taken as units were considered (*P *> 0.05).

## Discussion

In this study, 96 isolates belonging to the species *B. cenocepacia *IIIB and the BCC6 group, recovered from maize rhizosphere in Italy and Mexico, were characterized by using MLRT, in order to investigate the genetic diversity and relationships of bacteria associated with maize cultivated in geographically distant locations. Despite the clear relationship found between the geographic origin of isolates and grouping, identical RTs and closely related isolates were observed in geographically distant regions (Mexico and Italy). Two main complexes were identified following eBURST analysis, namely RT-4 for *B. cenocepacia *IIIB and RT-104 for BCC6. These two main clonal complexes included RTs shared by both Italian and Mexican maize rhizospheres, suggesting some mixing of the genotypes between the two continental regions and excluding the possibility of any kind of geographic subspeciation in the formation of these two complexes. At the genus and species level, many prokaryotes have a cosmopolitan distribution in their respective habitats and the same genotypes have often been identified in similar habitats in different geographic areas [40]. The wide geographic distribution and substantial capability of *Burkholderia *spp. to colonize diverse host plants was observed in distantly separated environments [21,24], as well as genetic identity between BCC isolates of clinical and environmental origins recovered from different countries has been proved [12]. Grouping isolates by eBURST analysis is useful to better evaluate the RTs distribution in natural population where highly similar RTs are found, i.e. to elucidate the meaning of the presence of closely related strains in geographically separated maize rhizospheres in respect to niche specificity and adaptation. Our finding that the majority of BCC6 isolates are part of RT-104-complex suggests that large networks of closely related BCC6 isolates colonize the maize rhizosphere of plants cultivated in Italy and Mexico. On the other hand, the presence of four clonal complexes and 12 singletons within the *B. cenocepacia *IIIB population suggests that maize rhizosphere is commonly colonized by well adapted *B. cenocepacia *IIIB clones rather than large networks of closely related isolates.

In spite of its lower discriminatory power in respect to MLST (restriction fragments *vs *sequences), MLRT provides useful data for typing and structure population investigations [26,28,32,35]. Previous MLST analyses performed on 26 Italian BCC isolates examined in the present work indicate a good correspondence between RTs and sequence types (STs) for certain isolates: i.e., three BCC6 isolates, typed by RT 34, had ST 127, and four isolates, typed by RT 81, had ST132 [20]. Conversely, MLST and MLRT data do not always match and the same ST for different RTs as well as different STs for the same RT were occasionally found [20]. Considering that MLRT and MLST do not rely on the same loci, we cannot strictly correlate our MLRT results with the MLST sequence database. Indeed, a previous study on *S. aureus *isolates [37] revealed that MLRT performed on the same seven loci used in MLST captures about 95% of the discrimination power of MLST, and demonstrated that MLRT approach represents a convenient alternative to MLST. The analyses of MLRT data using tools developed for MLST permit to assess clonality/recombination in our maize-rhizosphere populations. This is an important feature when assessing the risks for human health posed by opportunistic pathogens present in the natural environment. Bacterial population structures can vary from the extremes of strictly clonal to panmictic, with most populations occupying a middle ground where recombination is significant in the evolution but the emergence of epidemic clonal lineages can also occur [41-44]. The difference in the $I_{A}^{S}$ values between complete and corrected data sets (when the RTs are taken as units) suggests that both *B. cenocepacia *and BCC6 group have an epidemic population structure in which occasional clones emerge and spread. Both populations are recombining in the long term but a few RTs have recently become abundant and widespread [20,42]. Similar "epidemic" population structure has been observed in global collections of *B. cenocepacia *[32], and may occur continuously in microbial populations not affected by the severe selective constraints imposed by human activity [45]. The $I_{A}^{S}$ values calculated on a subset of isolates chosen on the basis of geographical origin evidenced a population structure different from that obtained considering the entire dataset. Concerning the BCC6 group, the Italian population behaved like the whole BCC6 population, showing linkage equilibrium only when RTs were taken as units (epidemic structure), while the Mexican population showed linkage equilibrium at all levels (freely recombining population structure). Regarding the *B. cenocepacia *IIIB populations, the Italian one was freely recombining, while the Mexican one had a clonal structure. Nevertheless, the $I_{A}^{S}$ values of the Mexican population are quite low, which may indicate that some recombination occurs. Recombination has had an important role in the long-term evolution of *B. cenocepacia *and it was also found among strains from different locations [20,32]. Most likely, the efficiency of genetic exchange mechanisms, due to BCC inherent genomic plasticity, together with ecological factors, play a crucial role.

The use of a common MLRT scheme for both *B. cenocepacia *IIIB and BCC6 group allowed to compare their genetic variability, relatedness, and population structure also at interspecific level. *B. cenocepacia *IIIB and BCC6 populations shared identical alleles but not the same RTs. In the UPGMA tree, where the genetic similarities between the restriction profiles of both *B. cenocepacia *IIIB and BCC6 group were represented, the isolates were grouped into two main clusters (clusters I and II) corresponding to their taxonomic status and eBURST clonal complexes; i.e., cluster I for *B. cenocepacia *IIIB and RT-4-complex, and cluster II for BCC6 group and RT-104-complex. Within each cluster, the occasional presence of few isolates belonging to the other BCC species is not surprising since BCC6 and *B. cenocepacia *IIIB are closely related, and indeed BCC6 was previously included in the *B. cenocepacia *species. UPGMA performed with only the isolates included in the RT-4 and RT-104 clonal complexes gave rise to a dendrogram showing two clusters exactly corresponding to them (data not shown), confirming the correspondence between eBURST and UPGMA grouping. Finally, the finding of a clear relationship between grouping and maize cultivar suggests that maize cultivars could influence rhizosphere bacterial diversity probably due to the different chemical composition of root exudates. In fact, it is well known that plant root bacterial communities are very sensitive to environmental conditions and are more strongly influenced by plant species and different cultivars rather than by other environmental factors such as soil type and agricultural practices [46-49].

## Conclusions

In conclusion, our data demonstrate a wide dispersal of certain *B. cenocepacia *IIIB and BCC6 isolates in Mexican and Italian maize rhizospheres. Despite the clear relationship found between the geographic origin of isolates and grouping, identical RTs and closely related isolates were observed in geographically distant regions. The differences in rhizosphere habitats and/or maize varieties between Italy and Mexico may result in certain selective pressure which may preferably promote some genotypes within each local microbial population, favouring the spread of a single clone above the rest of the recombinant population. Investigation of other important population genetic forces, such as gene flow and natural selection, with more extensive and/or focused sampling, would provide more insight into the spatial and temporal dynamics of BCC populations.

## Methods

### Bacterial isolation

A total of 31 *B. cenocepacia recA *lineage IIIB isolates (13 from Italian and 18 from Mexican maize-rhizosphere) and 65 BCC6 isolates (53 from Italian and 12 from Mexican maize-rhizosphere) were analysed.

Italian *B. cenocepacia *IIIB and BCC6 isolates investigated in this work represent a subsample of BCC populations recovered over a 8-year period (1995-2002) from the rhizosphere of different modern commercial varieties of maize cultivated in three fields located in different regions: S. Maria di Galeria, Rome (MC population), Pieve d'Olmi, Cremona (MVP population) and Dragoni, Caserta (MD population). Each bacterial population included distinct sub-populations recovered from the rhizosphere of different maize cultivars: MCII/MCIII in MC population, recovered in 1995 and 1997 from Fir and Pactol cultivars, respectively; MVPC1/MVPC2 in MVP population, recovered in 1996 from Airone and Goldiane cultivars, respectively; MDII/MDIII in MD population, recovered in 1996 and 2002 from Doge and Eleonora cultivars, respectively [[49-53], our unpublished data]. The majority of isolates were recovered by using the semi-selective PCAT medium [54], while MDIII isolates were selected from three different media (PCAT, TB-T or BAc, as indicated by the letters P, T or B, respectively) [21,53].

Mexican *B. cenocepacia *IIIB and BCC6 isolates investigated in this work belong to *Burkholderia *populations recovered in 2002 from the rhizosphere of maize plants cultivated in two sites located in the State of Morelos: Tetecala (MexII isolates from 57 to 264), where the modern commercial variety named Costeño mejorado was planted, and Amatlipac (MexII isolates from 815 to 1011), where the traditional maize variety named Criollo was planted. After 90-110 days of growth, 16 maize plants were randomly harvested in each site at a distance of 10 m between each other. Roots were excised from plants and loosely adhering soil was removed. The excised roots were randomly grouped into four samples, each comprising four root systems. Afterwards, each root sample was cut into small pieces (0.2-0.7 cm) and mixed thoroughly. Five grams of each mixture were suspended in 10 ml of potassium phosphate buffer (PPB 0.02 M, pH 6.8) added with 50 μl of Tween 80. Each root suspension was shaken by vortexing for 3 min at maximum speed. Samples were serially diluted in PBB and 100 μl of serial dilutions were plated on PCAT medium amended with 100 μg ml^-1 ^of cycloheximide (Sigma) to inhibit fungal growth. Plates were incubated at 29°C for 48 h. Single small colonies (diameter, about 1-2 mm), white or pale yellow with well-defined margins, were randomly picked up from the same dilution of root slurry sample, i.e. 1000-fold dilution from plates containing approximately 50-100 colonies. Isolates were subjected to single-colony isolation on the same medium and cryopreserved at -80°C in 30% glycerol until use.

### Isolate identification

Isolates were identified by means of *Hae*III *recA *restriction fragment length polymorphism (RFLP) and species-specific PCRs as previously reported [55]. RFLP profiles were compared with those of published reference strains as appropriate. All Italian isolates have been identified at the species level in previous works [19,20,22,52,53]. Fourteen Mexican isolates characterized by *recA *RFLP profile J' were identified as *B. cenocepacia *IIIB, while 12 Mexican isolates showing the *recA *RFLP profile AD were assigned to BCC6 group (present study). Two Mexican isolates with the RFLP profile I (which gave uncertain identification) and two Mexican isolates with RFLP profiles which were never recovered among BCC reference strains examined were assigned to *B. cenocepacia *IIIB by MLST analysis (Table 1) [22].

### MLRT characterization and data analysis

DNA preparation, PCR amplification of nearly complete sequence of five open reading frames of *recA, gyrB, fliC, cepIR *and *dsbA *genes, enzymatic restriction digests and separation of the resulting restriction fragments were performed as described previously [26]. Gel images were digitalized using GelDoc 2000 (Bio-Rad) and stored as TIFF files. Different restriction patterns for each locus were considered to represent separate alleles, and an arbitrary number was assigned to each allele. The different combinations of alleles for the five loci represented different allelic profiles. An arbitrary number [restriction type (RT)] was assigned to each allelic profile. The different restriction patterns found at each locus were analysed with DNA START-2 (Sequence Type Analysis and Recombination Test, version 2) software package http://pubmlst.org/software/analysis/start2/[56]. RT data sets were also analyzed using the eBURST (Based Upon Related Sequence Types) algorithm v3 http://eburst.mlst.net/. MLRT profiles were also analyzed by means of BioNumerics (Applied Maths) software 6.0. Cluster analysis was carried out on data defined as character type data. A similarity matrix was created by using the unweighted pair group method with arithmetic means algorithm (UPGMA) in order to assess the genetic relationships between the restriction profiles. The cophenetic correlation coefficient was used as a statistical method to estimate the error associated with dendrogram branches, while the Cluster Cutoff method was applied to define the most reliable clusters.

### Linkage disequilibrium analysis

The genetic diversity at individual loci (*h*), the mean genetic diversity (*H*_*mean*_) and the standardized index of association ($I_{A}^{S}$) were calculated using the LIAN version 3.5 software program (Department of Biotechnology and Bioinformatics University of Applied Sciences Weihenstephan; http://adenine.biz.fh-weihenstephan.de/cgi-bin/lian/lian.cgi.pl) [57]. The $I_{A}^{S}$, that is independent on the number of loci considered, was calculated as $I_{A}^{S}=\left[1∕\left(L-1\right)]\times [\left(V_{\text{D}}∕V_{\text{e}}\right)-1\right]$, where *V*_D _is the observed variance, *V*_e _is the variance expected for linkage equilibrium, and *L *is the number of loci analysed. The significance of linkage disequilibrium was tested by a parametric method [58] as implemented in LIAN 3.5.

## Competing interests

The authors declare that they have no competing interests.

## Authors' contributions

AB conceived and coordinated the study, and drafted the manuscript. BC carried out MLRT and linkage disequilibrium analyses. CC performed UPGMA analysis and prepared the manuscript's figures. SC performed eBURST analysis. LC participated in the design of the study and discussion of data. ST revised the manuscript. JCM contributed to the study design as well as was involved in the discussion of data and manuscript preparation. CD participated in discussion of data, in drafting and revising the manuscript. All authors read and approved the final manuscript.
